# Deacetylation of XBP1s by sirtuin 6 confers resistance to ER stress-induced hepatic steatosis

**DOI:** 10.1038/s12276-019-0309-0

**Published:** 2019-09-20

**Authors:** In Hyuk Bang, Oh Kwang Kwon, Lihua Hao, Dami Park, Myung-Ja Chung, Byung-Chul Oh, Sangkyu Lee, Eun Ju Bae, Byung-Hyun Park

**Affiliations:** 1 Department of Biochemistry, Chonbuk National University Medical School, Jeonju, Jeonbuk 54896 Republic of Korea; 2 College of Pharmacy, Kyungpook National University, Daegu, 41566 Republic of Korea; 3 Department of Pathology, Chonbuk National University Medical School, Jeonju, Jeonbuk 54896 Republic of Korea; 4 Department of Physiology, Gachon University College of Medicine, Incheon, 21999 Republic of Korea; 5 College of Pharmacy, Chonbuk National University, Jeonju, Jeonbuk 54896 Republic of Korea

**Keywords:** Translational research, Non-alcoholic fatty liver disease

## Abstract

The active spliced form of X-box-binding protein 1 (XBP1s) is a key modulator of ER stress, but the functional role of its post-translational modification remains unclear. Here, we demonstrate that XBP1s is a deacetylation target of Sirt6 and that its deacetylation protects against ER stress-induced hepatic steatosis. Specifically, the abundance of acetylated XBP1s and concordant hepatic steatosis were increased in hepatocyte-specific Sirt6 knockout and obese mice but were decreased by genetic overexpression and pharmacological activation of Sirt6. Mechanistically, we identified that Sirt6 deacetylated a transactivation domain of XBP1s at Lys257 and Lys297 and promoted XBP1s protein degradation through the ubiquitin-proteasome system. Overexpression of XBP1s, but not its deacetylation mutant 2KR (K257/297R), in mice increased lipid accumulation in the liver. Importantly, in liver tissues obtained from patients with nonalcoholic fatty liver disease (NAFLD), the extent of XBP1s acetylation correlated positively with the NAFLD activity score but negatively with the Sirt6 level. Collectively, we present direct evidence supporting the importance of XBP1 acetylation in ER stress-induced hepatic steatosis.

## Fatty liver disease: Regulatory protein link points to potential therapy

Activating a protein that regulates cellular health could protect against fat accumulation during the onset of non-alcoholic fatty liver disease (NAFLD). A high-fat diet disrupts the endoplasmic reticulum (ER), a cellular membrane network responsible for synthesizing and processing proteins and fats, and can lead to NAFLD development. Previous studies found that a protein called XBP1s activates fat-related genes during NAFLD. Byung-Hyun Park and Eun Ju Bae at Chonbuk National University in Jeonbuk, South Korea, and co-workers, recently demonstrated that high levels of a regulatory protein called Sirt6 limits liver inflammation and ER stress during high-fat diets. Now, in experiments on mouse models and human liver cells, Park’s team have shown that Sirt6 reduces liver ER stress by modifying XBP1s. Encouraging Sirt6 activation may help protect against NAFLD progression.

## Introduction

Nonalcoholic fatty liver disease (NAFLD) is a disorder characterized by hepatic fat accumulation (steatosis) that can progress to nonalcoholic steatohepatitis (NASH)^1^. During chronic high-calorie diet feeding, an increased demand for protein synthesis leads to the disruption of endoplasmic reticulum (ER) homeostasis, which is termed ER stress^2^. ER stress, in turn, induces the unfolded protein response (UPR) that directs unfolded or misfolded proteins to molecular chaperones to be refolded or degraded. In mammalian cells, the UPR is initiated by three ER stress sensor proteins: inositol-requiring enzyme 1α (IRE1α), protein kinase-like ER kinase (PERK), and activating transcription factor 6 (ATF6)^3^. Upon exposure to ER stressors, IRE1α undergoes dimerization and transautophosphorylation, which activates its endoribonuclease activity and cleaves XBP1 mRNA, leading to the translation of a spliced form of XBP1 (XBP1s)^4^. As a transcription factor, XBP1s upregulates the expression of specific genes involved in ER biogenesis and protein secretion, as well as molecular chaperones^5^.

Although ER stress has been implicated in a variety of liver diseases, including NAFLD^5–7^, how ER stress causes lipid accumulation in the liver remains largely unknown. Several mechanisms through which UPR molecules modulate ER stress-induced steatosis have been proposed. For example, a recent report showed that VLDL receptor upregulation by ER stress and subsequent lipid uptake mediate hepatic steatosis^8^. On the other hand, IRE1α has been shown to prevent ER stress-induced fatty liver development, as IRE1α knockout (KO) increased steatosis^9,10^. Notably, hepatocyte-specific deletion of XBP1s protected mice from hepatic steatosis and insulin resistance^11,12^, identifying XBP1s as a transcriptional activator of de novo lipogenic genes. Similarly, high-fat diet (HFD) feeding induces XBP1s in the liver, which mediates hepatic insulin resistance^13^. Thus, the downregulation of XBP1s seems to have potential as an approach for inhibiting ER stress-induced hepatic steatosis.

Sirtuins (from Sirt1 to Sirt7) have been implicated in NAFLD development under ER stress. Activation of Sirt1 by resveratrol prevents the development of NAFLD in HFD-fed mice through the suppression of ER stress^14^, suggesting that Sirt1 is a negative regulator of ER stress. Recent reports have shown that Sirt1 deacetylates XBP1s and inhibits its transcriptional activity, whereas acetyltransferase p300 increases the acetylation and protein stability of XBP1s^15^. Similarly, Sirt7 functions at the chromatin level to suppress ER stress and prevents NAFLD development^16^. Sirt6, a nuclear form of sirtuin, deacetylates the lysine residues of histone (H3K9, H3K18, and H3K56)^17–19^ and non-histone substrates^20–23^. Our group and others have found that mice lacking Sirt6 in hepatocytes develop NAFLD^24,25^ and that Sirt6 overexpression suppresses oxidative stress and inflammation in the liver under a high-calorie diet^24^. We also observed a decrease in Sirt6 protein levels in the liver of obese mice and NAFLD patients^24^; however, the role of Sirt6 in the ER stress response has not been studied.

In this study, we aimed to determine whether deletion, overexpression, or pharmacological activation of Sirt6 alters ER stress-induced hepatic steatosis and the associated pathology and to investigate the molecular mechanism of Sirt6 regulation of the UPR elicited by ER stress.

## Materials and methods

### Animals

Hepatocyte-specific Sirt6 KO mice (Sirt6^flox/flox^;albumin-Cre) were generated, as described previously^24^. Male Sirt6 KO mice and wild-type (albumin-Cre) littermates older than 6 weeks of age were fed a normal chow diet (NCD), a 60% HFD, or a 60% fat and 30% fructose diet (HFHF) for 10 weeks *ad libitum*. For the tunicamycin (Tm)-induced ER stress animal experiment, mice at 8–9 weeks of age were administered DMSO or Tm (Sigma-Aldrich, St. Louis, MO, USA) intraperitoneally at a dose of 1 mg/kg body weight. Sirt6 adenoviruses were injected intravenously *via* the tail vein two days before Tm treatment. All experimental procedures were approved by the Institutional Animal Care and Use Committee of Chonbuk National University (permit number: CBNU-2015-088).

### Human tissues

Human liver tissues were obtained from Chonbuk National University Hospital Biobank (CNUHB), with informed consent from the patients, and the study was approved by the Institutional Review Board of Chonbuk National University (permit number: T14-18)^24^. To assess morphological changes in the liver, we used the NAFLD activity score (NAS)^26^.

### Cell culture and transient transfection

For isolation of primary hepatocytes, mouse livers were perfused with collagenase type IV (Sigma-Aldrich), and hepatocytes were prepared as described previously^27^. The human hepatoma cell line HepG2 and human embryonic kidney cell line HEK293T were obtained from the American Type Culture Collection (Manassas, VA, USA). Exogenous proteins were expressed by transfecting HEK293T cells with 1 μg of XBP1s, Sirt6, Sirt6-H133Y (mSirt6), and p300 using Lipofectamine 3000 (Invitrogen, Carlsbad, CA, USA). For the XBP1s reporter gene assay, 2 μg of the ER stress response element (ERSE) luciferase promoter (Qiagen, Hilden, Germany) was used.

### Antibodies

Antibodies against the following proteins were used: Sirt6, p-Akt, Akt, Bax, cleaved caspase-3, Ac-K, Ac-H3K56, ATF4 (Cell Signaling, Beverly, MA, USA), GRP78, p-PERK, p-eIF2α, XBP1s, ubiquitin, ATF6, Sirt1 (Santa Cruz Biochemicals, Dallas, TX, USA), p-IRE1α (Abcam, Cambridge, UK), CHOP, lamin B, GAPDH (Bioworld Technology, St. Louis Park, MN, USA), Ac-H3K9, and β-actin (Sigma-Aldrich).

### Liquid chromatography (LC)-mass spectrometry (MS/MS) analysis

Immunoprecipitated proteins were visualized by staining SDS gels with colloidal Coomassie blue. After protein bands were excised from the gels, the proteins were subjected to in-gel digestion with trypsin. The tryptic peptides were analyzed by a high-resolution mass spectrometer (LTQ-Orbitrap Velos, Thermo Fisher Scientific, Waltham, MA, USA) coupled with nanoflow liquid chromatography (nano-LC) for acetylation, as described previously^28^.

### Site-directed mutagenesis

Acetylation mutants of XBP1s were generated using a site-directed mutagenesis kit (GeneAll, Seoul, Korea) by converting each lysine residue (K65, K146, K257, and K297) to arginine (codon change from AAG or AAA to AGG). Mutations were confirmed by DNA sequencing performed by Cosmogenetech (Seoul, Korea).

### Statistical analysis

The data are expressed as the mean ± standard error of the mean (SEM). Statistical comparisons were made using one-way analysis of variance followed by Fisher’s post hoc analysis. The significance of differences between two groups was determined using Student’s unpaired t-test. A *p* value of less than 0.05 was considered significant.

### Additional methods

Detailed methods and primer sequences are provided in the Supplementary Information.

## Results

### Severe hepatic steatosis in Sirt6 KO mice is associated with upregulation of the UPR

We and others have previously reported a reduction in the expression of Sirt6 in fatty livers^24,25^. To determine whether Sirt6 expression is altered by ER stress, we determined Sirt6 expression after treatment with ER stress inducers in hepatocytes. A decrease in the level of Sirt6 protein was observed after treatment with Tm, thapsigargin, and dithiothreitol, likely due to transcriptional suppression, as supported by the reduction in Sirt6 mRNA level after Tm treatment (Fig. S1a, b). Sirt6 downregulation by Tm was reversed by co-treatment with the chemical chaperone 4-phenylbutyrate (Fig. S1c). When mice were injected with Tm, Sirt6 expression in the liver decreased at 24 h and was restored at 48 h (Fig. S1d). At the same time, and consistent with previous reports^8^, hepatic triglyceride (TG) levels were highly increased, whereas the plasma TG level gradually decreased (Fig. S1e), and these changes were accompanied by increased expression of UPR signaling molecules, including XBP1s, p-PERK, p-eIF2α and ATF6 (p50) (Fig. S1f). These data imply that ER stress-induced hepatic steatosis may be linked to Sirt6 downregulation.

**Fig. 1:**
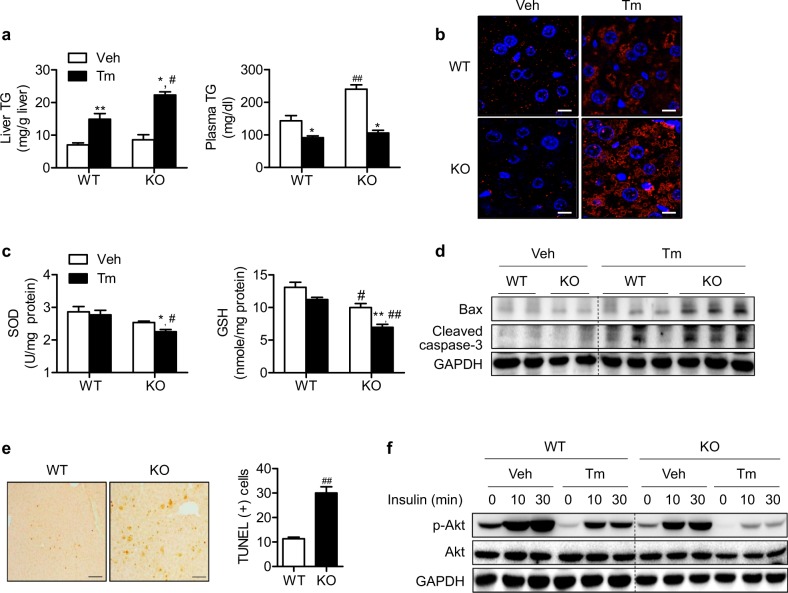
Enhancement of tunicamycin-induced ER stress response by Sirt6 deficiency. WT and hepatocyte-specific Sirt6 KO mice were treated with Tm (1 mg/kg) for 24 h. **a** Hepatic and plasma TG levels were determined (*n* = 4–5). **b** Perilipin immunostaining for hepatic lipid droplets. Bars = 10 μm. **c** WT and KO mice were treated with Tm (1 mg/kg) for 6 h, and superoxide dismutase (SOD) activity and glutathione (GSH) levels in liver tissues were determined by commercial assay kits (*n* = 4). **d** WT and KO mice were treated with Tm for 24 h, and the protein levels of Bax and cleaved caspase-3 in liver tissues were examined by western blotting. **e** Representative TUNEL staining of liver sections. Bars = 100 μm. The numbers of TUNEL-positive cells were counted and expressed as a percentage of the total hepatocytes (*n* = 4–6). **f** Primary hepatocytes were isolated from WT and KO mice and treated with Tm (2 μg/ml) for 16 h. Cells were stimulated with insulin (10 ng/ml) as indicated and immunoblotted with antibodies against p-Akt. The values are the mean ± SEM. *p < 0.05 and **p < 0.01 versus vehicle; ^#^*p* < 0.05 and ^##^*p* < 0^.^01 versus WT

To investigate the direct role of Sirt6 during the development of ER stress-induced hepatic steatosis, we compared the response of WT and KO mice to a Tm challenge. Sirt6 deficiency led to a robust increase in hepatic fat accumulation upon Tm administration as assessed by hepatic TG levels (Fig. 1a) and immunohistochemical staining for lipid droplets (Fig. 1b). Basal levels of plasma TG were significantly increased in KO mice, whereas Tm treatment suppressed plasma TG levels in WT and KO mice (Fig. 1a).

Because the hepatic UPR is also linked with oxidative stress, apoptosis, and insulin resistance^2^, we next examined the effects of Sirt6 deficiency on these parameters. Tm treatment significantly decreased superoxide dismutase activity and glutathione levels in the livers of KO mice compared with those in WT mice (Fig. 1c). Tm-induced apoptosis and insulin resistance, which were assessed by western blotting for pro-apoptotic molecules or Akt phosphorylation and TUNEL staining, were more evident in Sirt6 KO livers than in WT livers (Figs. 1d–f, S2).

### XBP1s activation is a major determinant of the increase in the Tm-induced UPR in Sirt6 KO mice

**Fig. 2:**
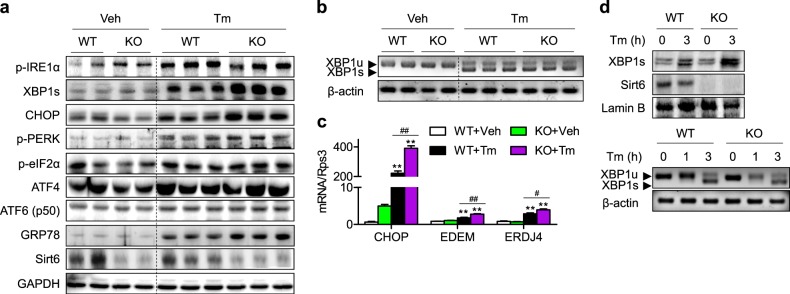
Activation of the XBP1s pathway by Sirt6 deficiency. WT and KO mice were treated with Tm (1 mg/kg) for 24 h. **a** ER stress markers in the livers were examined by western blotting. **b** Spliced and unspliced XBP1 mRNA levels were detected using RT-PCR. **c** The mRNA levels of CHOP, EDEM, and ERDJ4 were analyzed by real-time RT-PCR (*n* = 4–6). **d** Primary hepatocytes isolated from WT and KO mice were treated with Tm (2 μg/ml), and the protein levels of XBP1s in the nuclear fraction and XBP1 mRNA splicing were determined by western blotting and RT-PCR, respectively. The values are the mean ± SEM. **p < 0.01 versus vehicle; ^#^*p* < 0.05 and ^##^*p* < 0.01 versus WT

To identify the UPR transducers responsible for hepatic steatosis in Sirt6 KO mice, we carefully investigated the activation status of three transducers of ER stress signaling. Although Tm-stimulated phosphorylation of IRE1α was not different between genotypes, expression levels of XBP1s and its target genes (CHOP, EDEM, and ERDJ4) were greatly increased in Sirt6 KO mice (Figs. 2a, c, S3). Phosphorylation levels of PERK, its downstream molecule p-eIF2α and GRP78 were also higher in KO mice than in WT mice, while ATF6 (p50) expression was not changed (Figs. 2a, S3). Because XBP1s expression is largely regulated by XBP1 mRNA splicing, we next measured XBP1 mRNA splicing by RT-PCR. As shown in Fig. 2b, Tm-induced XBP1 mRNA splicing was not changed between genotypes, which was in line with the findings of no changes in IRE1α phosphorylation in KO mice (Figs. 2a, S3). The Sirt6 deficiency-induced increase in XBP1s levels upon Tm stimulation with no difference in XBP1 mRNA splicing was further confirmed in primary hepatocytes (Fig. 2d).

### Sirt6 overexpression alleviates steatosis, apoptosis, and insulin resistance in the liver

**Fig. 3:**
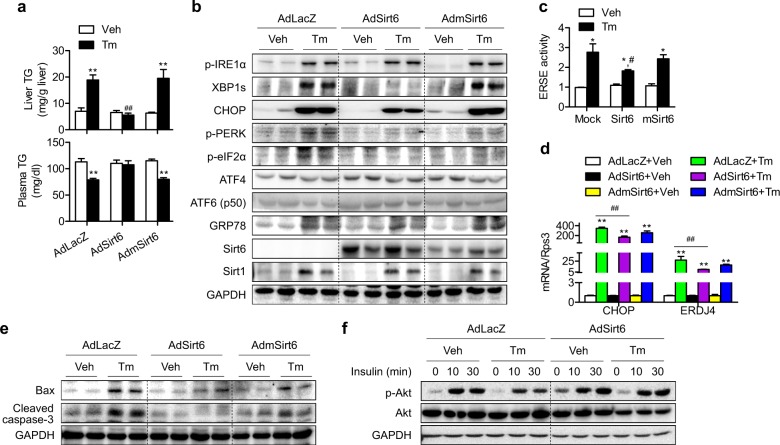
Suppression of the tunicamycin-induced ER stress response by Sirt6. **a** Mice were injected intravenously with 1 × 10^9^ pfu of AdLacZ, AdSirt6, or AdmSirt6 and then treated with Tm (1 mg/kg) for 24 h. Hepatic and plasma TG levels were analyzed (*n* = 4–6). **b** ER stress markers in the livers were examined by western blotting. **c** HepG2 cells were treated with Tm (2 μg/ml) for 20 h, and ERSE luciferase activity in the cell lysates was assayed (*n* = 4). **d**, **e** mRNA levels of CHOP and ERDJ4 (*n* = 4) and protein levels of Bax and cleaved caspase-3 in liver tissues were analyzed by real-time RT-PCR and western blotting, respectively. **f** HepG2 cells were treated with Tm (2 μg/ml), and insulin-stimulated Akt phosphorylation was determined. The values are the mean ± SEM. *p < 0.05 and **p < 0.01 versus vehicle; ^#^*p* < 0.05 and ^##^*p* < 0^.^01 versus AdLacZ or mock

Next, we aimed to assess whether Sirt6 overexpression protects the liver from ER stress. To this end, C57BL/6 mice were injected with AdLacZ, AdSirt6, or AdmSirt6, followed by Tm exposure for 24 h. Tm-induced hepatic TG accumulation and lower plasma TG levels were completely abolished in mice injected with AdSirt6 but not AdmSirt6 (Fig. 3a). Among the three pathways of UPR, XBP1s- and PERK-dependent pathways were significantly attenuated by hepatic overexpression of Sirt6 (Figs. 3b, S4a). The ERSE reporter assay results indicated that Tm-induced transactivation of XBP1s was significantly diminished by overexpression of Sirt6 but not mSirt6 (Fig. 3c). In agreement with this finding, the XBP1s targets CHOP and ERDJ4 were also significantly suppressed by Sirt6 overexpression (Fig. 3d), confirming the downregulation of the XBP1s signaling pathway by Sirt6. Because Tm-induced hepatocyte apoptosis was increased in Sirt6 KO mice (Fig. 1d, e), we next investigated whether Sirt6 overexpression protects against apoptotic cell death. Western blotting and TUNEL staining demonstrated that apoptosis was almost completely blocked by Sirt6 overexpression (Figs. 3e, S4b, S5). Finally, Tm treatment of HepG2 cells reduced insulin-stimulated Akt phosphorylation, whereas Sirt6 overexpression fully restored hepatic insulin sensitivity (Figs. 3f, S4c).

### Sirt6 decreases the stability of the XBP1s protein

**Fig. 4:**
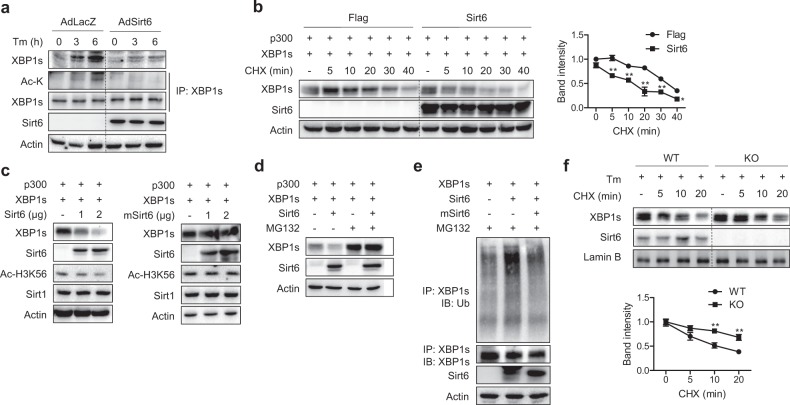
Acceleration of proteasome-dependent XBP1s degradation by Sirt6. **a** HepG2 cells transduced with AdLacZ or AdSirt6 were treated with Tm (2 μg/ml) for the indicated time periods, and the protein levels of total and acetylated XBP1s were analyzed. To pull down equal amounts of XBP1s for each lane in which the endogenous XBP1s protein levels were variable, we used a minimal amount of anti-XBP1s antibody for IP. **b** HEK293T cells transfected with p300 and XBP1s were treated with cycloheximide (CHX, 20 μg/ml) for the indicated times, and the relative protein levels of XBP1s were compared (*n* = 3). **c**, **d** After HEK293T cell transfection as indicated with or without 2 μM MG132, the protein levels of XBP1s were analyzed. **e** After transfection with the indicated plasmids, HEK293T cells were immunoprecipitated with anti-XBP1s antibodies and immunoblotted with anti-ubiquitin antibodies. **f** Primary hepatocytes from WT or Sirt6 KO mice were treated with Tm (2 μg/ml) with or without CHX for the indicated times. Relative protein levels of nuclear XBP1s were compared by western blotting (*n* = 3). The values are the mean ± SEM. *p < 0.05 and **p < 0.01 versus Flag or WT

We next investigated the molecular mechanism of XBP1s reduction by Sirt6, with a focus on its deacetylase activity. When HepG2 cells transduced with control or Sirt6 adenovirus were treated with Tm, the expression and acetylation of XBP1s were increased in the control group but not in Sirt6-overexpressing cells, implying that Sirt6 deacetylates XBP1s (Fig. 4a). Because the acetylation status of XBP1s has been reported to affect its stability^15^, we examined XBP1s stability in HEK293T cells transfected with constructs carrying p300 or XBP1s with or without Sirt6 and then chased the level of XBP1s after treatment with the protein synthesis inhibitor cycloheximide (CHX). CHX treatment gradually decreased the level of XBP1s with a faster rate of disappearance in cells overexpressing Sirt6 (Fig. 4b), indicating that Sirt6 reduces the stability of the XBP1s protein. Indeed, ectopic expression of Sirt6 dose-dependently decreased the total protein level of XBP1s in HEK293T cells co-expressing p300 and XBP1s, but deacetylase-inactive mSirt6 had no effect (Fig. 4c). Additional results showed that Sirt6-mediated XBP1s degradation was abolished in the presence of MG132, a proteasome inhibitor (Fig. 4d), indicating that Sirt6 induces proteasome-dependent degradation of XBP1s. Accordingly, Sirt6 overexpression increased the ubiquitination of XBP1s (Fig. 4e). Lastly, we repeated the CHX chase assay in primary hepatocytes isolated from WT or Sirt6 KO mice, and the results clearly indicated that Sirt6 deficiency increased the stability of the XBP1s protein (Fig. 4f). These findings suggest that Sirt6, which is reliant on its deacetylase enzymatic activity, causes the proteasomal degradation of XBP1s.

### Sirt6 deacetylates XBP1s at Lys65, Lys146, Lys257, and Lys297

**Fig. 5:**
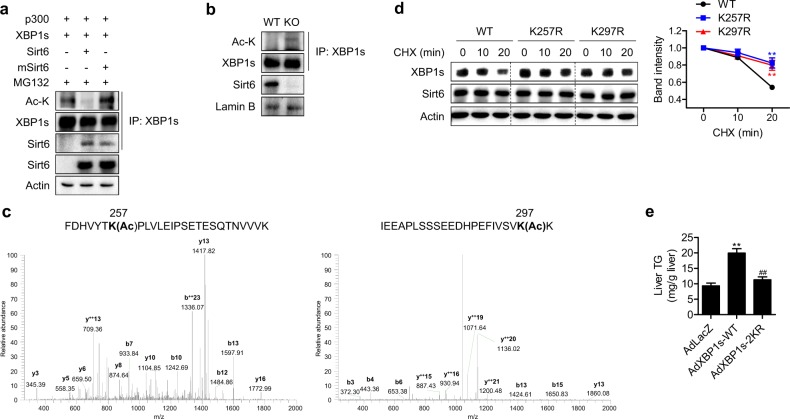
Identification of deacetylation sites in XBP1s. **a** HEK293T cells were transfected with the indicated plasmids, and total cell lysates were immunoprecipitated with anti-XBP1s antibodies and immunoblotted with anti-Ac-K and anti-Sirt6 antibodies. **b** Primary hepatocytes isolated from WT and KO mice were treated with Tm (2 μg/ml) and subjected to a co-immunoprecipitation assay for XBP1s deacetylation. **c** HEK293T cells were transfected with XBP1s with p300 or XBP1s with p300 and Sirt6. The acetylation of XBP1s was determined using LC-MS/MS analysis after in-gel digestion. MS/MS spectra of acetylated XBP1s peptides containing Lys257 (left) and Lys297 (right). **d** HEK293T cells were transfected with wild type (WT) or mutant XBP1s (K257R and K297R) and then treated with CHX (20 μg/ml) for the indicated times. Relative protein levels of XBP1s were compared (*n* = 3). **e** C57BL/6 mice were injected intravenously with 1 × 10^9^ pfu of AdLacZ, AdXBP1s-WT, or AdXBP1s-2KR, and hepatic TG levels were analyzed (*n* = 4–5). The values are the mean ± SEM. *p < 0.05 and **p < 0.01 versus WT or AdLacZ; ^##^*p* < 0.01 versus AdXBP1s-WT

Our finding that XBP1s acetylation was reduced in cells overexpressing Sirt6 was confirmed by additional experiments. We co-expressed XBP1s and p300 with either Sirt6 or mSirt6 in HEK293T cells and measured the acetylation level of XBP1s. When the immunoprecipitates of XBP1s were immunoblotted with an anti-acetyl lysine (Ac-K) antibody, robust acetylation of XBP1s was detected in control cells, but this acetylation was decreased by Sirt6, and catalytically inactive mSirt6 had no effect (Fig. 5a). To further validate these results, we compared the acetylation of XBP1s in primary hepatocytes isolated from WT and Sirt6 KO mice. The results showed an increase in XBP1s acetylation in KO hepatocytes (Fig. 5b). Together, these results suggest that XBP1s is a direct deacetylation substrate of Sirt6.

Next, we sought to identify the deacetylation sites of XBP1s. HEK293T cells were transfected with p300 with or without Sirt6, and XBP1s immunoprecipitates were analyzed by LC–MS/MS. Compared to cells transfected with p300 acetyltransferase only, four lysine residues (K65, K146, K257, and K297) were identified as deacetylated in cells overexpressing Sirt6 (Figs. 5c, S6a, Table S1). To determine whether the half-life of the XBP1s protein is increased when deacetylation at these residues is prevented by the presence of Sirt6, we substituted each residue with arginine (K65R, K146R, K257R, and K297R). We expressed either WT or mutated XBP1s along with Sirt6 and then repeated the CHX chase experiment. The stability of XBP1s was significantly increased with K257R and K297R but not with K65R and K146R compared to that of WT XBP1s (Figs. 5d and S6c, d), indicating that Sirt6 deacetylation of K257 and K297 mediates XBP1s protein degradation. Next, to assess whether acetylation of XBP1s plays a role in hepatic lipid accumulation, we injected C57BL/6 mice with adenoviruses carrying either XBP1-WT or double-mutant XBP1s (2KR, K257/297R). As shown in Fig. 5e, hepatic TG levels in mice with AdXBP1s-WT, but not with AdXBP1s-2KR, were higher compared to those with AdLacZ. Increases in steatosis induced by XBP1s-WT were further confirmed by perilipin immunostaining (Fig. S7a). Furthermore, XBP1s-WT, but not XBP1s-2KR, induced hepatic apoptosis and insulin resistance in mice and hepatocytes, respectively (Fig. S7b, S7c). These results collectively indicate that XBP1s acetylation at Lys257 and Lys297 is critical in XBP1s-mediated hepatic steatosis, apoptosis, and insulin resistance.

### Pharmacological activation of Sirt6 attenuates Tm-induced ER stress and fat accumulation in the liver

**Fig. 6:**
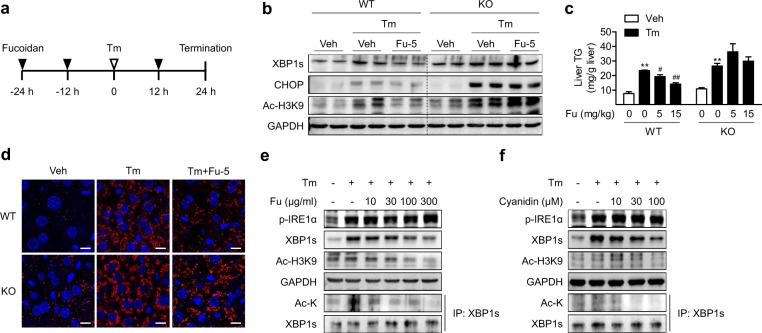
Prevention of tunicamycin (Tm)-induced fat accumulation by pharmacological Sirt6 activation. **a** WT and KO mice were treated with fucoidan (5 or 15 mg/kg) three times *via* oral gavage and injected with Tm (1 mg/kg) for 24 h. **b** ER stress-associated markers in the livers were examined by western blotting. **c**, **d** Hepatic TG accumulation was analyzed using a specific TG assay kit (*n* = 3–4) or perilipin immunostaining. Bars = 10 μm. **e**, **f** HepG2 cells were pretreated with either fucoidan (Fu) or cyanidin at the indicated concentrations prior to Tm (2 μg/ml) treatment for 6 h. Protein levels of total and acetylated XBP1s were determined by western blotting. The values are the mean ± SEM. **p < 0.01 versus vehicle; ^#^*p* < 0.05 and ^##^*p* < 0.01 versus Tm

Recently, Rahnasto-Rilla et al^29^. identified that fucoidan, which is a natural sulfated polysaccharide extracted from brown seaweed, is a Sirt6 activator. Therefore, we aimed to evaluate the effects of fucoidan on ER stress-mediated hepatic steatosis. WT and Sirt6 KO mice were administered fucoidan via oral gavage three times, as depicted in Fig. 6a, and liver tissue was analyzed. We first confirmed that fucoidan acted as a Sirt6 activator in mice by finding decreased H3K9 acetylation in WT livers, which was not observed in KO livers (Fig. 6b). Fucoidan administration effectively suppressed Tm-induced XBP1s and CHOP expression and TG accumulation in the livers of WT mice but not in the livers of Sirt6 KO mice (Fig. 6b–d), indicating that alleviation of the ER stress response by fucoidan was mediated by Sirt6. The results obtained from further experiments *in vitro*, in which HepG2 cells were treated with fucoidan or a structurally distinct Sirt6 activator cyanidin^30^, were in good agreement with the *in vivo* data in that treatment with these agents decreased the levels of total and acetylated XBP1s in a concentration-dependent manner (Fig. 6e, f). Again, successful activation of Sirt6 by fucoidan and cyanidin was indicated by a reduction in Ac-H3K9.

### The XBP1s signaling pathway is activated in HFD- and HFHF-fed Sirt6 KO mice

**Fig. 7:**
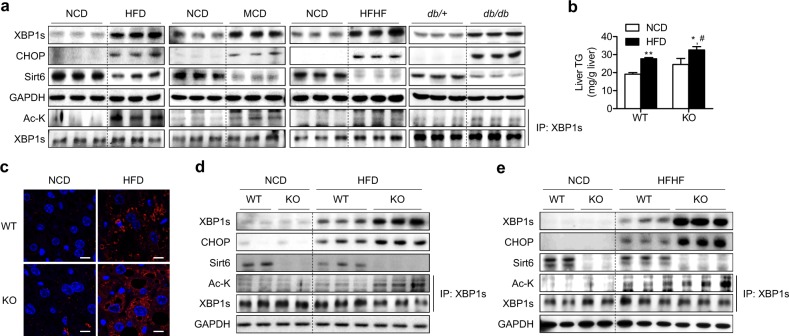
Activation of the XBP1s pathway in diet-induced animal models of NAFLD by Sirt6 deficiency. **a** Protein levels of XBP1s and CHOP were analyzed in 10-week HFD-fed mice, 4-week MCD-fed mice, and 10-week HFHF-fed mice and in 12-week-old db/db mice. **b**, **c** Hepatic TG levels and perilipin immunostaining for lipid droplets were determined. Bars = 10 μm. **d**, **e** Protein levels of XBP1s and CHOP were compared in NCD-, HFD-, or HFHF-fed WT and KO mice. The values are the mean ± SEM. *p < 0.05 and **p < 0.01 versus NCD; ^#^*p* < 0.05 versus WT

To determine whether the XBP1s pathway is altered by nutrient status, we analyzed hepatic XBP1s and CHOP expression in various NAFLD animal models with low Sirt6 expression. Compared to control mice, dietary (i.e., HFD, MCD, or HFHF) or genetic (db/db) mice models of NAFLD had markedly elevated levels of both total and acetylated XBP1s and CHOP protein in liver (Fig. 7a). The impact of Sirt6 deficiency was further investigated in mice fed a HFD or HFHF to physiologically mimic NAFLD development in humans. When fed a HFD, hepatic steatosis was enhanced in KO mice, as identified by hepatic TG levels (Fig. 7b) and immunostaining for hepatic lipid droplets (Fig. 7c). Similar to the results of the Tm study, the XBP1s pathway was upregulated even more in the livers of HFD- and HFHF-fed Sirt6 KO mice than in the livers of WT mice (Fig. 7d, e).

### XBP1 acetylation is increased in patients with NAFLD

**Fig. 8:**
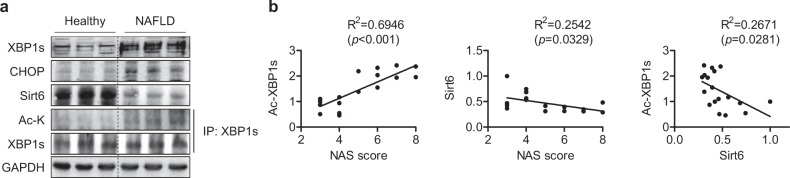
Increased XBP1s acetylation in the liver of NAFLD patients. **a** Expression of total and acetylated XBP1s and Sirt6 in livers from healthy subjects and NAFLD patients was analyzed by western blotting. **b** The correlation between NAFLD activity score (NAS), Ac-XBP1s and Sirt6 (*n* = 18)

To provide clinical relevance, we compared the XBP1s acetylation status in patients with NAFLD and healthy subjects. Total and acetylated XBP1s levels were noticeably higher in the liver tissues of NAFLD patients than in those of healthy controls (Fig. 8a). Consistent with our previous report^24^, Sirt6 protein levels were decreased in NAFLD patients. The most important finding was that the NAFLD activity score (NAS) was highly positively correlated with the levels of acetylated XBP1s but negatively correlated with Sirt6 expression (Fig. 8b). Moreover, Ac-XBP1s was negatively correlated with Sirt6 expression, indicating that Sirt6 directly mediates XBP1s deacetylation. These findings suggest that the reduced expression of Sirt6 and concordant increases in acetylation, and thus XBP1s expression, contribute to the disease progression of NAFLD in patients.

## Discussion

This study was designed to address the hypothesis that Sirt6 ameliorates hepatic ER stress by deacetylating XBP1s to improve fat accumulation, apoptosis, and insulin resistance in hepatocytes. The plausibility of this hypothesis was derived from the previous finding that prolonged and unmitigated ER stress is tightly linked to NAFLD^7,31^ and that hepatic insulin resistance and steatosis in NAFLD are aggravated by Sirt6 deficiency^24,25^. Here, we showed for the first time that hepatocyte-specific Sirt6 deficiency exacerbated the XBP1s pathway of the UPR, at least in part, by regulating the protein stability of XBP1s *via* its deacetylation (Fig. S8). Additionally, we demonstrated that Sirt6 overexpression or pharmacological activation counteracted Tm-induced ER stress to alleviate hepatic steatosis and associated pathologies.

In this study, several lines of evidence suggest that Sirt6 has a protective role against ER stress. First, Sirt6 KO mice exhibited an aggravated ER stress response compared with WT mice after Tm administration or high-calorie diet feeding. Among the three pathways of UPR signaling, activation of XBP1s, and to a lesser extent the PERK pathway, but not ATF6, had a significant impact on the ER stress response in Sirt6 KO hepatocytes. Supporting this notion, the protein levels of XBP1s and its target genes CHOP, EDEM, and ERDJ4 were greatly increased in the livers of KO mice. In contrast, genetic overexpression and pharmacological activation of Sirt6 in hepatocytes were sufficient to reduce the activation of the XBP1s pathway, both *in vitro* and *in vivo*.

Second, Tm-stimulated hepatic steatosis was increased in Sirt6 KO mice but decreased by Sirt6 augmentation, which was assessed by perilipin immunostaining and the TG content of the liver. Because a variety of molecules in the liver are supposed to mediate the dysregulation of lipid metabolism under ER stress, we sought to determine which genes or pathways were involved in the attenuation of steatosis by Sirt6. Unlike previous reports, lipogenic gene (e.g., SREBP-1c, fatty acid synthase, stearoyl-CoA desaturase, and diacylglycerol acetyltransferase 2) and very low-density lipoprotein receptor expression levels were not increased by Tm stimulation. Furthermore, the Tm-induced change in the expression of PPARα, which is essential for fatty acid oxidation, was modest. The expression of all these genes was preserved upon Sirt6 KO or overexpression. These results imply that ER stress inducers, or the resulting adaptive response, regulate hepatic lipid metabolism in a way that cannot be simply attributed to a single metabolic pathway.

Third, Sirt6 deficiency resulted in impaired insulin signaling and increased apoptosis under ER stress, whereas Sirt6 overexpression and Sirt6 activation improved hepatic insulin resistance and apoptosis. Previous studies have shown that reducing ER stress with chemical chaperones improves insulin resistance and cell injury^7,32,33^. It is likely that all three UPR signaling pathways are fundamental to ER stress-induced apoptosis. Among others, CHOP, whose induction depends on XBP1s, ATF4, and ATF6, is known to promote apoptotic cell death partly by blocking the anti-apoptotic effect of Bcl2^34^. In the current study, Sirt6 strongly inhibited XBP1s activation and, to a lesser extent, PERK phosphorylation; both of these effects might contribute to marked CHOP inhibition and subsequent hepatocyte apoptosis. In addition, ER stress induces hepatic insulin resistance mainly through lipotoxicity and XBP1s. The inhibition of XBP1s activation by siRNA has been found to protect against palmitate-induced downregulation of Akt phosphorylation^35^. Thus, diminished XBP1s activation by Sirt6 might contribute to the restoration of hepatic insulin sensitivity in our study.

Finally, total and acetylated XBP1s protein levels were significantly higher in the liver tissues of NAFLD patients than in those of healthy controls, suggesting an inhibitory role of Sirt6-mediated deacetylation of XBP1s in NAFLD pathogenesis. We found here that Sirt6 deficiency led to strong activation of the XBP1s pathway under ER stress. Because the production of XBP1s is largely dependent on XBP1 mRNA splicing, which is regulated by IRE1α^36^, we assessed the levels of p-IRE1α as well as XBP1u and XBP1s mRNA and found that there were no differences between the genotypes. Combining these data with the finding that deacetylase-inactive mSirt6 had no effect on XBP1s levels, we hypothesized that the increase in XBP1s in Sirt6 KO mice may be mediated by post-translational modification, i.e., deacetylation. Although the acetylation/deacetylation status has been shown to significantly affect the stability and activity of the XBP1s protein^9,15,32^, the acetylation of which specific lysine residues plays a critical role remains unknown. Through LC-MS/MS analysis and a subsequent functional study, we identified that Sirt6 deacetylates XBP1s at Lys65, Lys146, Lys257, and Lys297 and that the deacetylation of the last two residues leads to its degradation through a proteasome-mediated pathway. Our results indicate a reduction in Sirt6 expression in NAFLD and other conditions associated with ER stress, and the consequent increase in XBP1s activity *via* acetylation-dependent protein stabilization leads to enhanced steatosis, insulin resistance, and injury in the liver. Indeed, in human liver, the expression of acetylated XBP1s is highly correlated with NAS in NAFLD patients with reduced Sirt6 expression.

In light of the fact that acetyl-CoA is abundant as an intermediary product of glucose catabolism, histone acetylation has emerged as a link between epigenetic regulation and NAFLD development and has thus gained increasing attention over the past decade as a therapeutic option against NAFLD. For instance, hepatocyte-specific HDAC3 deletion caused steatosis^37,38^, and this was due to enhanced hepatic de novo lipogenesis after release from protein complexes with transcription repressors. Although our current study focused on the deacetylation of a nonhistone protein, i.e., XBP1s, by Sirt6, all of these studies support the notion that (de)acetylation of histone or nonhistone substrates may provide an important targeting strategy against hepatic fat accumulation and associated pathologies. Altogether, Sirt6 activation may play a protective role against ER stress-induced NAFLD progression.

## Supplementary information

Suppl Information
