# Supplementary material for: Deacetylation of XBP1s by sirtuin 6 confers resistance to ER stress-induced hepatic steatosis
Source: Exp Mol Med. 2019 Sep 20;51(9):1–11. doi: 10.1038/s12276-019-0309-0 (PMC6802632; doi:10.1038/s12276-019-0309-0)
Supplement: Supplementary file 1 — Suppl Information [file 12276_2019_309_MOESM1_ESM.pdf]

Deacetylation of XBP1s by sirtuin 6 confers resistance to ER stress-induced hepatic steatosis

In Hyuk Bang, Oh Kwang Kwon, Lihua Hao, Dami Park, Myung-Ja Chung, Byung-Chul Oh, Sangkyu Lee, Eun Ju Bae, and Byung-Hyun Park

Contents

1. Supplementary materials and methods
2. Supplementary figures
3. Supplementary tables

1. Supplementary Materials and Methods

Animals

Male C57BLS/J and db/db mice were purchased from Samtako (Osan, Korea) and The Jackson Laboratory (Bar Harbor, ME, USA), respectively. Diets (NCD, MCD, HFD, and HFHF) were purchased from Research Diet (New Brunswick, NJ, USA). C57BLS/J mice were fed with MCD diet for 4 weeks, HFD for 10 weeks, or HFHF diet for 10 weeks *ad libitum*. Male KO mice and age-matched wild-type (albumin-Cre) littermates were fed a NCD, HFD, or HFHF diet (Research Diet, New Brunswick, NJ, USA) for 10 weeks *ad libitum*. Mice were housed in standard laboratory conditions (23±1°C, 40-60% relative humidity, and a 12-h light-dark cycle) in a barrier facility with laminar flow cabinets. For the tunicamycin (Tm)-induced endoplasmic reticulum (ER) stress animal experiment, mice at 8–9 weeks of age were administered intraperitoneally with DMSO or Tm (Sigma-Aldrich, St. Louis, MO, USA) at a dose of 1 mg/kg body weight. Sirt6 adenoviruses were given two days before Tm treatment. Mice were sacrificed at 6 or 24 h after Tm treatment under anesthetic conditions, and livers were immediately placed in fixative (10% formalin solution in 0.1 M PBS) or snap-frozen in liquid nitrogen before storage at −80°C. All animal experiments were performed in accordance with the Guide for the Care and Use of Laboratory Animals, published by the US National Institutes of Health (NIH Publication No. 85-23, revised 2011). The current study protocol was approved by the Institutional Animal Care and Use Committee of Chonbuk National University (permit number: CBNU-2015-088).

Preparation of recombinant adenovirus

Adenoviruses expressing Sirt6 (AdSirt6), a catalytically inactive mutant Sirt6-H133Y (AdmSirt6) and β-galactosidase (AdLacZ) were prepared as described previously[39]. To prepare XBP1s-WT or XBP1s-2KR-expressing adenovirus, mouse XBP1s complementary DNA (cDNA) was inserted into pAdTrack-CMV vector (Addgene, Watertown, MA, USA). The pAdTrack-CMV-XBP1-WT or 2KR plasmid was cloned into the pAdEasy-1 vector (Agilent, Santa Clara, CA, USA). The plasmid was linearized with Pac I and was transfected into 293A cells using Lipofectamine 2000 (Invitrogen). The experimental details were followed the protocol[40].

Biochemical analysis

Plasma levels of triglyceride (TG) were determined using a plasma TG assay kit (Asan Pharmaceutical, Seoul, Korea). To quantify liver TG, liver tissues were homogenized and extracted in a mixture of chloroform, methanol, and DW (2/1/1 ratio). The TG concentration was expressed as milligrams of TG per 100 mg of liver tissue. To analyze the enzymatic activity of superoxide dismutase, liver tissues were suspended in 10 mM phosphate buffer (pH 7.4), mixed with ice-cold 5% metaphosphoric acid solution, and homogenized. Homogenates were centrifuged at 5,000 × *g* for 10 min. Enzyme activity in the supernatant was determined using commercial assay kits (Enzo Life Sciences, Plymouth Meeting, PA, USA). Glutathione was analyzed using specific ELISA kits (Enzo Life Sciences).

Histology

Liver histological sections (4 μm) were cut from formalin-fixed paraffin-embedded tissue blocks. Immunofluorescence staining was performed using the DAKO Envision system

(DAKO, Carpinteria, CA, USA). After deparaffinization, tissue sections were immunostained with antibodies against anti-perilipin2 (Progen Biotechnik, Heidelberg, Germany). Perilipin 2 staining were analyzed by confocal microscope installed in the Center for University-Wide Research Facilities (CURF) at Chonbuk National University. Peroxidase activity was detected with 3-amino-9-ethyl carbazole. Apoptosis in the liver was determined by TUNEL staining.

Cell culture and transient transfection

The human hepatoma cell line HepG2 and human embryonic kidney cell line HEK293T were obtained from American Type Culture Collection (Manassas, VA, USA). Cells were grown in DMEM supplemented with 10% fetal bovine serum, 2 mM glutamine, 100 U/ml penicillin and 100 μg/ml streptomycin. To express exogenous proteins, HEK293T cells were mock transfected or transfected with 1 μg of XBP1s, Sirt6, Sirt6-H133Y (mSirt6), and p300 using Lipofectamine 3000 (Invitrogen, Carlsbad, CA, USA). For the XBP1s reporter gene assay, 2 μg of ERSE promoter luciferase (Qiagen, Hilden, Germany) were used. After 48 h, cells were harvested in reporter lysis buffer (Promega, Madison, WI, USA). Luciferase activity was measured using a Dual-Luciferase Reporter Assay (Promega) by Lumat LB 9507 (Berthold, Bad Wildbad, Germany).

Western blotting and co-immunoprecipitation

Liver homogenates or cell lysates (20 μg) were separated by 10 or 12% SDS-PAGE and transferred to PVDF membranes. After blocking with 5% skim milk, the blot was probed with primary antibodies Sirt6 (D8D12), p-Akt (D9E), Akt (2H10), Bax, cleaved caspase-3 (5A1E), Ac-K, Ac-H3K56, ATF4 (D4B8) (Cell Signaling, Beverly, MA, USA), GRP78 (H-129), p-

PERK, p-eIF2α, XBP1s (M-186), ubiquitin (P4D1), ATF6 (H-280), Sirt1 (H-300) (Santa Cruz Biochemicals, Dallas, TX, USA), p-IRE1α, (abcam, Cambridge, UK), CHOP (K121), lamin B (L75), GAPDH (A531) (Bioworld Technology, St Louis Park, MN, USA), Ac-H3K9, or β-actin (ac-15) (Sigma-Aldrich). For co-immunoprecipitation, 500 μg of nuclear protein precleared with protein G-agarose was incubated with minimal amounts of anti-XBP1s overnight at 4°C, and then with protein G-agarose at 4°C for 2 h. Blots were probed with primary antibodies against Ac-K (Cell Signaling), XBP1s, or ubiquitin and signals were detected with a Las-4000 imager (GE Healthcare Life Science, Pittsburgh, PA, USA).

Mass spectrometry analysis

Immunoprecipitated proteins were visualized by staining SDS gels with colloidal Coomassie blue. Proteins were excised from the gel bands, applied with reduction and alkylation using dithiothreitol (DTT) and iodoacetamide (IAA), respectively, digested by trypsin at a ratio of 1:50 (w/w), and then the mixture was incubated at 37°C overnight[41]. After tryptic peptides were desalted by C_{18} ziptip (Millipore, Bedford, MD, USA), they were analyzed in an LTQ Orbitrap Velos mass spectrometer (Thermo Fisher Scientific, Waltham, MA, USA) connected with a nano-LC system (SCIEX, Framingham, MA, USA). The peptides were separated by a home-made capillary column (150 mm length × 75 mm internal diameter) with Jupiter C_{12} resin (Phnomenex, 4 μm particle size, 90 Å pore diameter). We used 60 min gradient from 2–20% solvent B (99.9% acetonitrile and 0.1% formic acid) to solvent A (5% acetonitrile in 0.1% formic acid) at a flow rate of 300 nl/min. To identify acetylated lysine, the LTQ-Orbitrap Velos was operated in the top 20 data-dependent mode using the following parameters: 1.8 kV of nano-electrospray voltage; 300-1,800 m/z range; 60,000 resolution for

full MS; fragmented by collision-induced dissociation (CID) with 28% normalized collision energy (NCE) and 1.7 m/z of isolation width; dynamic exclusion: 30 s; repeat count: 1. MS/MS spectra were searched using Mascot (version 2.3, Matrix Science Ltd, London, UK). Search parameters were set as a precursor mass tolerance of 20 ppm and 0.5 Da for precursor and fragment, respectively, variable modification of acetylation at protein *N*-term and lysine residues and oxidation to methionine, fixed modification of carbamidomethyl on cysteine, and trypsin as the digestion enzyme. The search results were filtered by a Mascot peptide score of more than 20 and all lysine-acetylated peptides were manually verified as in previous paper[42]. The detected Ac-K sites were confirmed to be substrates of Sirt6 or not by relative quantitative evaluation. The modified and unmodified peptides were scanned by MS/MS using the targeted data-dependent mode (Table S1). The relative abundance of Ac-K sites was determined by the ratio of the number of MS/MS spectra for peptides with the Ac-K residue *vs* the number of MS/MS spectra for the unmodified peptide corresponding to the same site.

Real-time quantitative RT-PCR (qPCR) and Xbp-1 splicing assay

Total RNA was extracted from frozen liver tissues or primary hepatocytes using an RNA Iso kit (TaKaRa, Tokyo, Japan). First-strand cDNA was generated using the random hexamer primer provided in the first-strand cDNA synthesis kit (Applied Biosystems, Foster City, CA, USA). qPCR reactions comprised a final volume of 10 μl, containing 10 ng of reverse-transcribed total RNA, 200 nM of forward and reverse primers and PCR master mixture. qPCR was performed in 384-well plates using an ABI Prism 7900HT Sequence Detection System (Applied Biosystems). For XBP-1 splicing, reverse transcription and PCR were performed using a One-Step RT-PCR kit (Invitrogen). PCR fragments were separated by electrophoresis

on 2% agarose gels, followed by staining with ethidium bromide. The primers were designed using qPrimerDepot (http://mouseprimerdepot.nci.nih.gov, Table S2).

2. Supplementary Figures

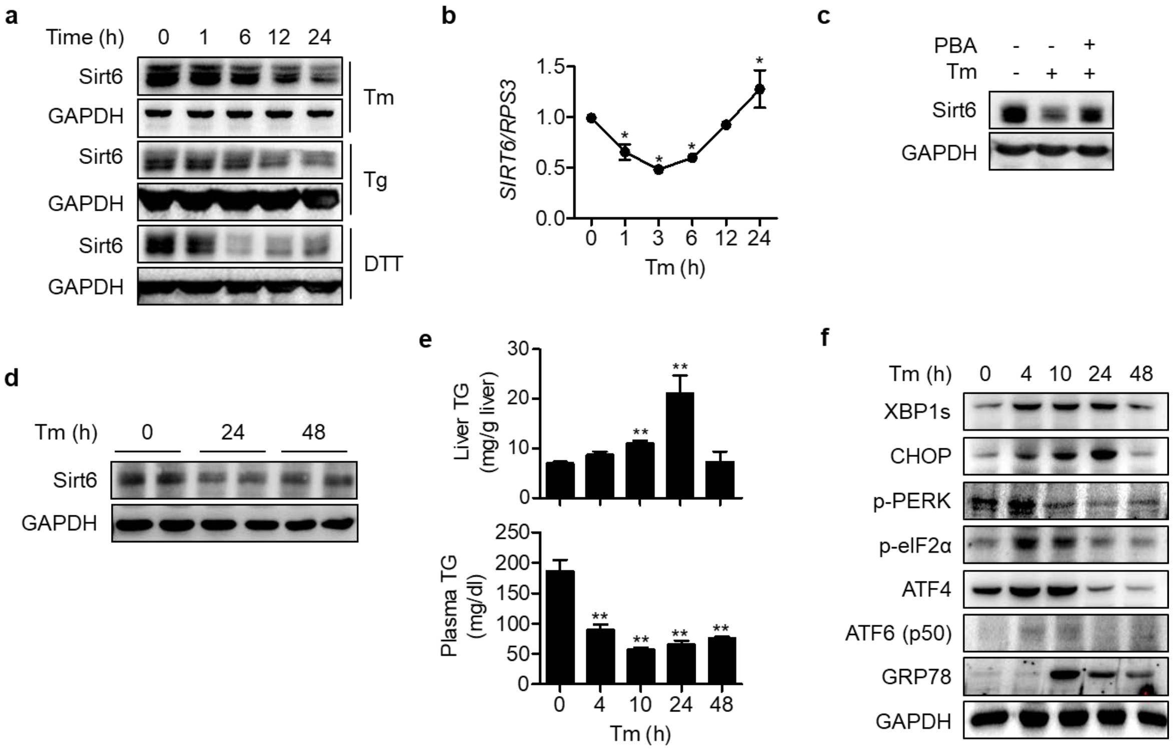

Figure S1. Sirt6 expression and hepatic TG accumulation in response to ER stress. a–c HepG2 cells were treated with tunicamycin (Tm, 2 μg/ml), thapsigargin (Tg, 100 nM), dithiothreitol (DTT, 100 μM), or Tm with 4-phenylbutyrate (PBA, 1 mM) and Sirt6 protein and mRNA levels were analyzed (n=4). **d–f** C57BL/6 mice were injected with Tm (1 mg/kg) intraperitoneally and protein levels of Sirt6, hepatic and plasma levels of TG, and ER stress markers were determined (n=3). Values are the mean±SEM. *, p<0.05 and **, p<0.01 versus control.

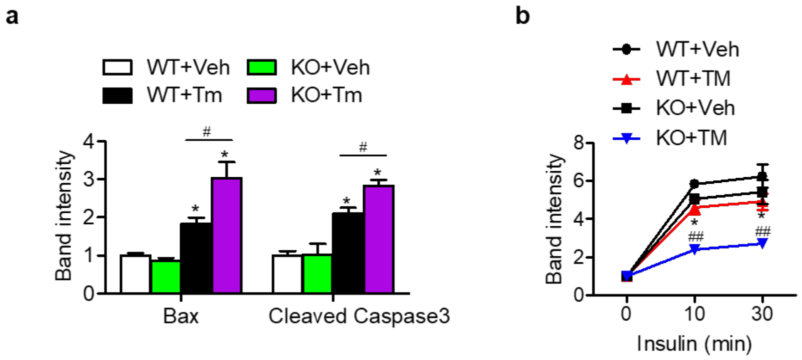

Figure S2. Densitometric analysis of Western blots. a The band intensities shown in Fig. 1d were quantified by densitometry (n=3). **b** The band intensities shown in Fig. 1f were quantified by densitometry (n=3). The band intensity of each protein was normalized to the intensity of GAPDH band. Values are the mean±SEM. *, p<0.05 and **, p<0.01 versus Vehicle; $^{\#}$, p<0.05 and $^{\#\#}$, p<0.01 versus WT.

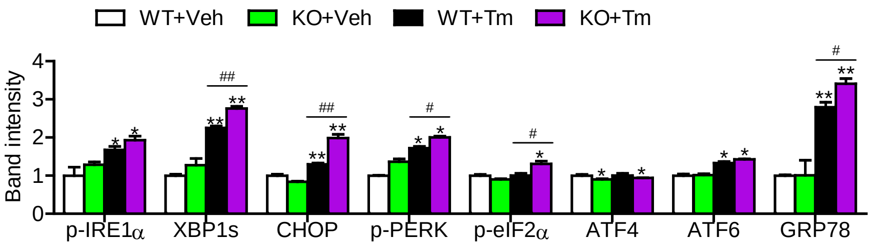

Figure S3. Densitometric analysis of Western blots. The band intensities shown in Fig. 2a were quantified by densitometry (n=3). The band intensity of each protein was normalized to the intensity of GAPDH band. Values are the mean±SEM. *, p<0.05 and **, p<0.01 versus Vehicle; #, p<0.05 and ##, p<0.01 versus WT.

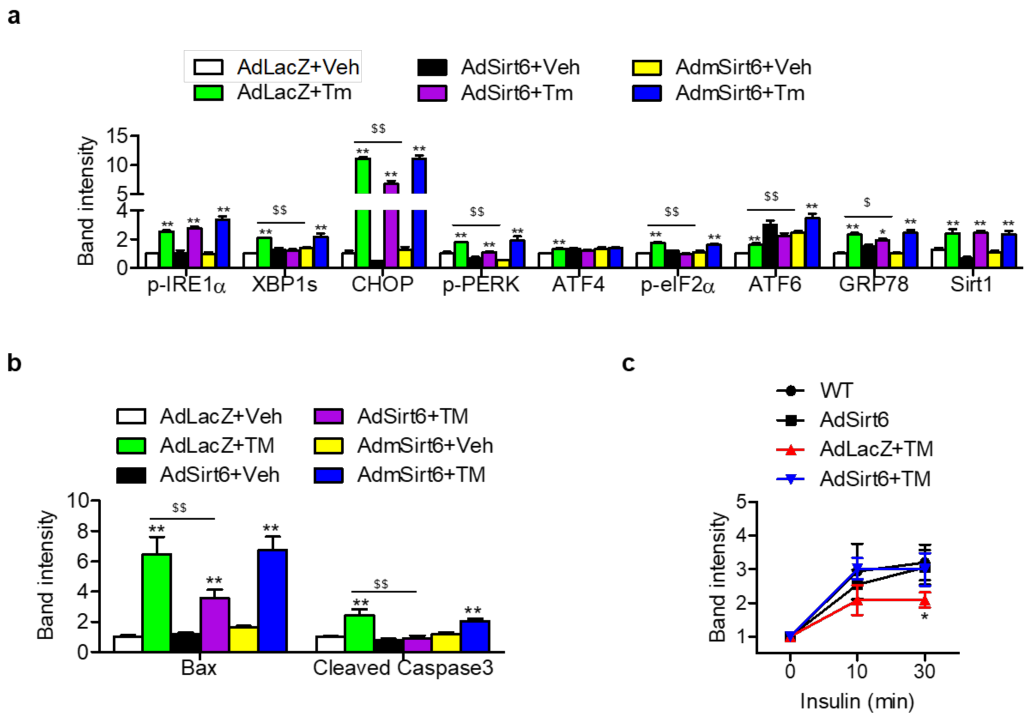

Figure S4. Densitometric analysis of Western blots. a The band intensities shown in Fig. 3b were quantified by densitometry. **b** The band intensities shown in Fig. 3e were quantified by densitometry. **c** The band intensities shown in Fig. 3f were quantified by densitometry. The band intensity of each protein was normalized to the intensity of GAPDH band. Values are the mean±SEM (n=4). *, *p*<0.05 and **, *p*<0.01 versus Vehicle; $^{\$}$, *p*<0.05 and $^{\$\$}$, p<0.01 versus AdLacZ.

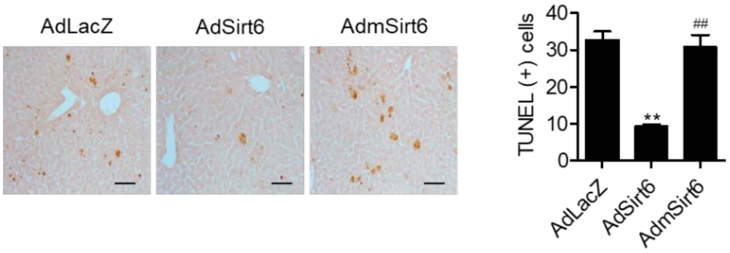

Figure S5. Suppression of tunicamycin-induced apoptosis by Sirt6. Mice were transduced with control or Sirt6 adenovirus and treated with Tm (1 mg/kg) for 24 h. Representative TUNEL stains of liver sections. The numbers of TUNEL-positive cells were counted and expressed as a percentage of total hepatocytes (n=4). Values are the mean±SEM. **, $p<0.01$ versus AdLacZ; $^{\#\#}$, $p<0.01$ versus AdSirt6.

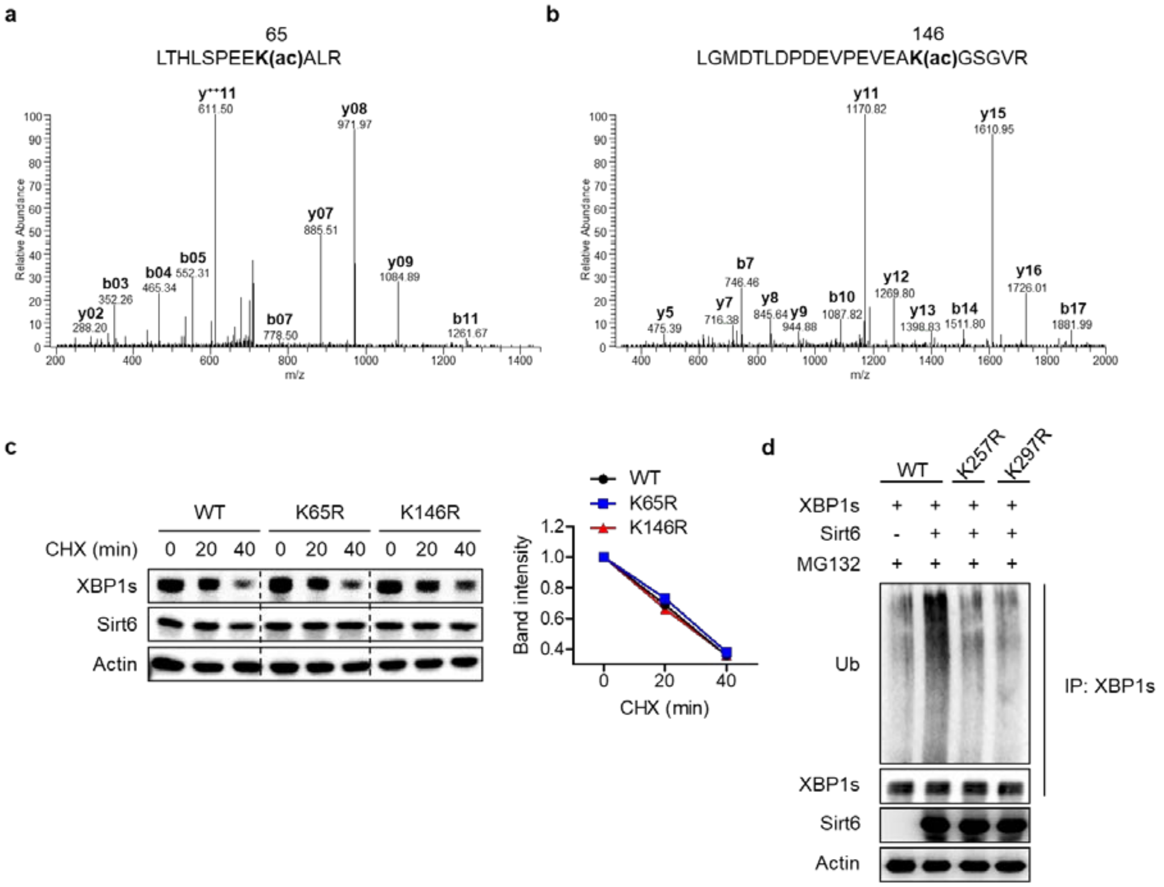

Figure S6. Protein stability of XBP1s-K65R and XBP1s-K146R. a, b MS/MS spectra of acetylated XBP1s peptides containing Lys65 and Lys146. **c** HEK293T cells were transfected with p300, wild or mutant XBP1s, and Sirt6 and then treated with CHX (20 μg/ml) for the indicated time periods. Relative protein levels of XBP1s were compared (n=3). **d** After transfection with wild (WT) or mutant XBP1s (K257R and K297R), HEK293T cells were immunoprecipitated with anti-XBP1s antibodies and immunoblotted with anti-ubiquitin antibodies. Values are the mean±SEM.

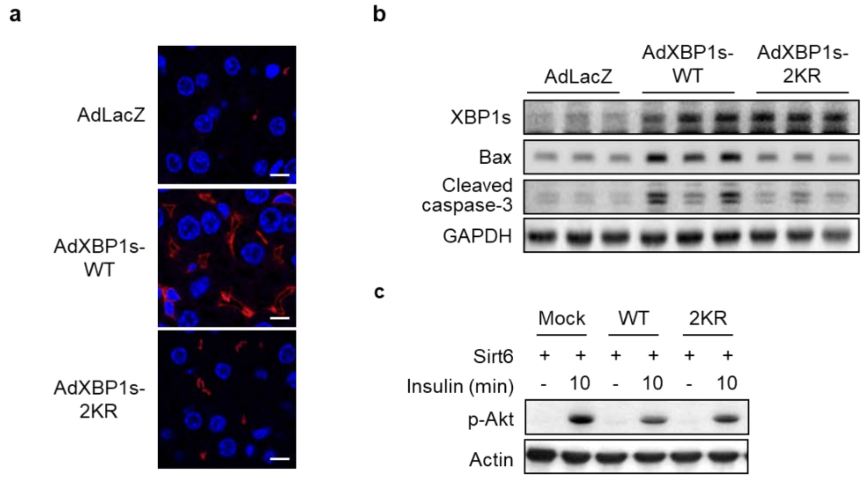

Figure S7. Suppression of hepatic TG accumulation and apoptosis by XBP1s-2KR mutant. **a** Perilipin immunostaining for hepatic lipid droplets. Bars=10 µm. **b** Bax and cleaved caspase-3 in liver tissues were analyzed by Western blots. **c** HepG2 cells were transfected with wild (WT) or K257/297R double mutant XBP1s (2KR), and insulin-stimulated Akt phosphorylation was determined.

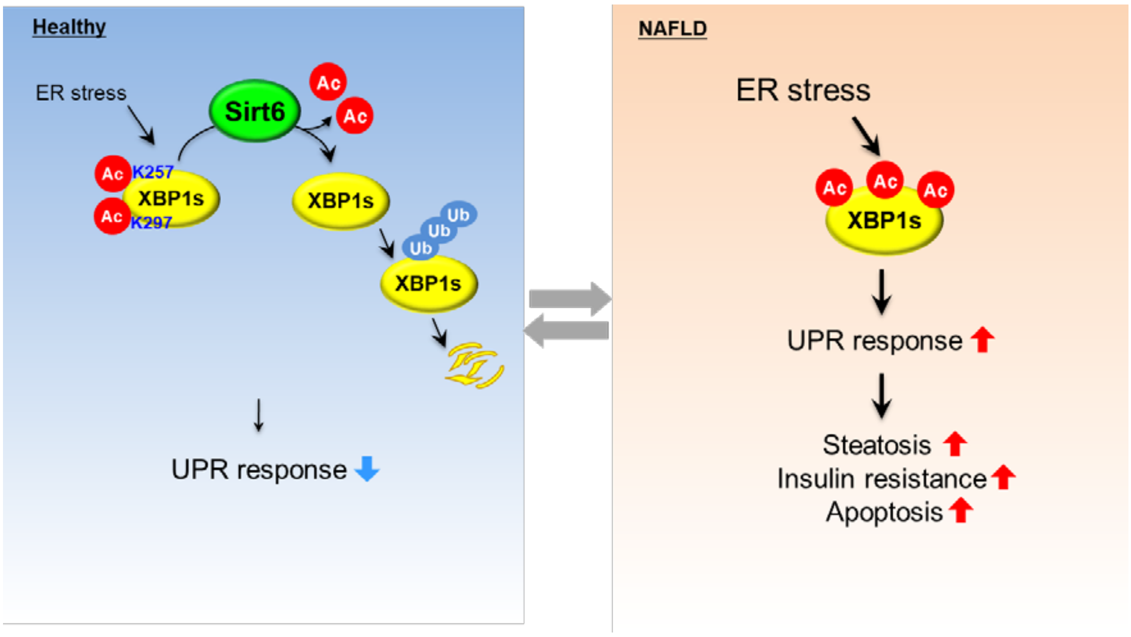

Figure S8. Proposed summary.

3. Supplementary Tables

Table S1. List of precursor ions of targeted peptides bearing Ac-K

Ac-K site	Sequence	Precursor ion (m/z, z=3)	Relative area (Kac/non-Kac)	
			Flag	Sirt6
K65	LTHLSPEE**K**(Ac)ALR<br>LTHLSPEE**K**	479.2683<br>351.8574	0.2384	0.1083
K146	LGMDTLDPDEVPEVEA**K**(Ac)GSGVR<br>LGMDTLDPDEVPEVEA**K**	786.0494<br>619.9644	0.0224	0.0105
K257	FDHVYT**K**(Ac)PLVLEIPSETESQTNVVVK<br>FDHVYT**K**PLVLEIPSETESQTNVVVK	1005.5289<br>991.5254	0.0314	0.0101
K297	IEEAPLSSSEEDHPEFIVSV**K**(Ac)K<br>IEEAPLSSSEEDHPEFIVSV**K**	838.0866<br>781.3848	0.1625	0.0293

Precursor ion of the acetylated tryptic peptides of XBP1s corresponding to residues 57–68 (LTHLSPEEKALR), 130–151 (LGMDTLDPDEVPEVEAKGSGVR), 251–276 (FDHVYTKPLVLEIPSETESQTNVVVK), and 277–298 (IEEAPLSSSEEDHPEFIVSVKK), which were identified from immunoprecipitated XBP1s in HEK293T cells.

Table S2. Sequences and accession numbers for primers (forward, FOR; reverse, REV)

Gene	Sequences for primers	Accession No.
SIRT6	FOR: CCCACGGAGTCTGGACCAT<br>REV: CTCTGCCAGTTTGTCCCTG	NM_001193285
CHOP	FOR: TGTATCCAAGGCATCAACCA<br>REV: GACGAGCTCAACCCCATCTA	NM_001290183
GRP78	FOR: AGTCCAGCAATAGTGCCAGC<br>REV: AAGGAGACTGCTGAGGCGTA	NM_001163434
EDEM	FOR: TGTATCCAAGGCATCAACCA<br>REV: GACGAGCTCAACCCCATCTA	NM_138677
ERDJ4	FOR: GGCGTGCAGTTTTGAATTTG<br>REV: CAAACTCAGCCCGACACATA	NM_013760
XBP1	FOR: AACTCCAGCTAGAAAATCAGC<br>REV: CCATGGGAAGATGTTCTGGG	NM_001271730.1
RPS3	FOR: GGCAGTGTGACAGGTTGAAG<br>REV: ATCAGAGAGTTGACCGCAGTTG	NM_008084
